# Yoga Practitioners Uniquely Activate the Superior Parietal Lobule and Supramarginal Gyrus During Emotion Regulation

**DOI:** 10.3389/fnint.2018.00060

**Published:** 2018-12-04

**Authors:** Katie P. Wadden, Nicholas J. Snow, Peder Sande, Sian Slawson, Tom Waller, Lara A. Boyd

**Affiliations:** ^1^Graduate Program in Rehabilitation Sciences, University of British Columbia, Vancouver, BC, Canada; ^2^Graduate Program in Neuroscience Sciences, University of British Columbia, Vancouver, BC, Canada; ^3^Whitespace™ Innovation Team, lululemon athletica, Vancouver, BC, Canada; ^4^Department of Physical Therapy, University of British Columbia, Vancouver, BC, Canada; ^5^Centre for Brain Health, University of British Columbia, Vancouver, BC, Canada

**Keywords:** yoga, functional magnetic resonance imaging, heart rate variability, emotion regulation, physical activity

## Abstract

Chronic stress contributes to both mental and physical illness. A high prevalence and cost of stress-related illnesses North America warrants investigation into alternative or complementary therapies which may help reduce adverse reactions to stressful stimuli. Emotion regulation is the process of monitoring and adjusting emotional responses to environmental stimuli and stressors. Individuals who participate in physical activity are less likely to have adverse responses to potentially stressful situations, potentially due to adaptions in emotion regulation. Yoga is a form of physical activity involving stretching exercises and meditation, that may lessen individuals’ levels of stress and anxiety and improve emotion regulation. High-frequency heart rate variability (HF-HRV) is considered a measure of parasympathetic nervous system (PNS) activity during the emotion regulation. Measuring HRV and brain activity using functional magnetic resonance imaging (fMRI) offers a useful, noninvasive approach to evaluating “neurovisceral” components of emotion regulation. We aimed to determine whether yoga practitioners (YP) exhibit different patterns of brain activation compared to recreational athletes (RA) without current yoga experience, while viewing emotionally arousing visual stimuli. Our secondary aim was to examine potential differences across groups in HRV throughout the presentation of these stimuli. Analysis of fMRI data during exposure to emotion-evoking (EE) stimuli revealed that the YP group activated two unique brain areas, namely the superior parietal lobule and the supramarginal gyrus. These areas have been associated with attentional awareness and reduced egocentric bias, processes that have been implicated in emotion regulation by others. The RA group activated the inferior middle frontal cortex, an area associated with cognitive reappraisal during emotion regulation. The YP group also demonstrated a trend towards a higher ratio of low- to high-frequency HRV compared to the RA group. The present findings support the presence of experience-dependent neurovisceral mechanisms associated with emotion regulation. Individuals who practice yoga regulate their neurovisceral responses to potentially stressful external stimuli in a different manner than recreational athletes who do not engage in yoga practice. The present study had a small sample size (RA: *n* = 12; YP: *n* = 19), which should be taken into account when interpreting the results.

## Introduction

Stress has been implicated as a substantial contributor to both mental and physical illness, and can compromise psychological well-being (Schmidt et al., 2008; Yamawaki et al., 2012). People who experience a high degree of chronic stress are more vulnerable to serious chronic illnesses such as depression, anxiety, type-II diabetes mellitus and cardiovascular disease (Schmidt et al., 2008; Yamawaki et al., 2012). Due to the high prevalence of psychiatric disorders in North America, and their associated costly treatments (e.g., antidepressant medications and psychotherapy; Chisholm et al., 2016), alternative or complementary therapies have received increasing consideration (Asher et al., 2017). Physical activity interventions have a medium-term (3–12 months) antidepressant effect, as improved physical fitness is believed to reduce psycho-social stress responses (Krogh et al., 2015). This reduction may facilitate an individual’s capacity to cope with stress (Scully et al., 1998).

Yoga is a type of physical activity with growing popularity in North America, and is defined as “a practice of gentle stretching exercises for breath control and meditation as a mind-body intervention” (Ernst, 2006). During yoga-based interventions, individuals are taught to be aware of bodily sensations, feelings, emotions, or thoughts (Brown and Ryan, 2003; Keng et al., 2011). A key element is a state of mindfulness, which is believed to be a key mechanism through which yoga contributes to a reduction in the stress response (Shelov et al., 2009; Keng et al., 2011). In this manner, yoga, while being a physical practice, could have application as a cognitive tool to combat stress-related risks to individuals’ health status, through differences in emotion regulation strategies (Ortner et al., 2007; Chiesa and Serretti, 2011; Shennan et al., 2011; Chiesa et al., 2012). The mechanisms that contribute to improving emotion regulation strategies in response to physical activity participation occur both at the psychological and neurophysiological levels. Currently, the optimal type of physical activity to protect against psychological stress, the onset of depression, or recurrence of depressive symptoms is unknown (Rebar et al., 2015). A greater understanding into the mechanisms underlying the benefits of different forms of physical activity will aid in the overall understanding of how to optimize activity programming to counter psychological stress.

Emotion regulation is the process of monitoring and adjusting emotional responses to environmental stimuli, that is controlled by the autonomic nervous system (ANS; Thayer and Lane, 2000). Individuals with greater emotion regulation capabilities are less likely to have an adverse response to a threatening, or potentially stressful, situation (Yamawaki et al., 2012). Emotional responses are modulated by inhibition from the parasympathetic nervous system (PNS), which down-regulates activity of the sympathetic nervous system (SNS), and can modify interactions between specific brain regions during situations requiring emotion regulation (Lane et al., 2009; Thayer et al., 2012). High-frequency heart rate variability (HF-HRV) provides a non-invasive measure of PNS activity, and in combination with measures of brain activity can serve as an index of the “neurovisceral” components of PNS-modulated emotion regulation of stress (Thayer and Lane, 2000; Lane et al., 2009). HRV is a biomarker for stress, and influenced by a network of brain areas that directly regulate the activity of the heart, through the vagus nerve (Thayer et al., 2012). To date, HRV has been used as a biomarker for emotion regulation in studies investigating the effects of yoga and aerobic exercise-based interventions (Satyapriya et al., 2009; Kouidi et al., 2010; Nagendra et al., 2015). However, studies investigating the outcomes of yoga interventions on stress and emotion are typically brief and do not take into account alternative treatments or lifestyle comparisons (Brown and Ryan, 2003; Peng et al., 2004; Satyapriya et al., 2009; Keng et al., 2011; Li and Goldsmith, 2012). As such, there is a gap in the literature on the longer-term effects of yoga training (i.e., over a time-course of several years) on key neural components of emotion regulation, in comparison to other lifestyle factors, including regular (non-yoga-based) physical activity.

Presently, there are few magnetic resonance imaging (MRI)-based studies specific to individuals who practice yoga (Froeliger et al., 2012; Villemure et al., 2014, 2015). One recent study showed a relationship between years of yoga practice, minutes practiced per week, and gray matter volume in regions of the brain attributed to distinct components of yoga including posture, breathing, and meditation (Villemure et al., 2015). It is also well known that regular physical activity affects the structure the of brain including the hippocampus and prefrontal cortex, which are involved in memory, as well as executive function and mood (Erickson et al., 2014) functional MRI (fMRI) experiments show that physical activity also impacts brain activity during cognitive task performance (Colcombe et al., 2004) and in resting-state networks (Voss et al., 2010).

To date no research has investigated the relationships between emotion regulation, long-term yoga practice, PNS modulation of HRV and brain activation. Thus, the present study considered the effects of yoga practice on neurovisceral components (HRV and fMRI) of emotion regulation, using a cross-sectional sample of yoga practitioners (YP) and individuals who engage in regular physical activity but not yoga. Our principal aim was to determine whether individuals who practice yoga show a different pattern of brain activity when exposed to emotional visual stimuli, compared to individuals who are physically active but not engaged in yoga. Our secondary aim was to examine whether HRV differed across these groups. We exposed YP, and recreational athletes (RA) who did not practice yoga, to film clips containing previously-validated emotional content (Schaefer et al., 2010; Dunn and Hoegg, 2014) and simultaneously measured patterns of brain activity (using fMRI) and HRV [using electrocardiogram (ECG)] during the viewing of these film clips. We hypothesized that during observation of emotional stimuli YP participants would demonstrate unique patterns of brain activity in regions that have been implicated in emotion regulation, as compared to RA participants. We also hypothesized that YP participants would exhibit greater HF-HRV, compared to RA participants, during the emotional stimuli. Finally, we conducted a series of exploratory correlational analyses, between brain areas of significant activation, and HF-HRV during the emotional stimuli, and behavioral measures (metabolic equivalents of task (METs) of yoga per week and dispositional mindfulness) in the YP group. The latter analyses were based on evidence for an experience-dependent relationship between yoga practice and neurophysiology measures (Villemure et al., 2014, 2015), as well as evidence for a link between an individuals’ level of mindfulness, emotion regulation and neurophysiology (Hölzel et al., 2011; Zeidan et al., 2011), and could be used in generating hypotheses for future work.

## Materials and Methods

The present study was carried out in accordance with the recommendations of the Tri-Council Policy Statement, approved by the University of British Columbia (UBC) Clinical Research Ethics Board, as well as the UBC MRI Research Centre. All participants provided written and oral informed consent, in accordance with the principles of the Declaration of Helsinki. Participants were recruited using posters displayed throughout the University of British Columbia campus and surrounding community of Vancouver, British Columbia. For the YP group, additional recruitment emails were sent to local yoga studios in the Vancouver, British Columbia area.

### Participants

Thirty-one participants, at least 19 years of age (range = 19–60 years) were recruited for this study: 19 YP (*M* = 35.89, *SD* = 11.51; Female = 5) and 12 RA (*M* = 32.58, *SD* = 9.13; Female = 16). All participants were free of contraindications to MRI (i.e., free from implanted metal or electrical devices; not pregnant; no concurrent psychiatric or neurodegenerative disorders). Participants were excluded if they had a recent history of substance abuse or were taking prescription medication known to alter ANS activity (e.g., β-blockers). Prior to undergoing imaging procedures, all participants completed several questionnaires for the purposes of informing physical activity levels, personality characteristics, and yoga experience (see “Questionnaires” section); these questionnaires were used to characterize the study sample, and were also intended for use in exploratory correlational analyses (see “Statistical Analyses” section).

Individuals in the YP group reported practicing yoga for at least one to three 30- to 60-min sessions per week, and had accumulated at least 6 months of yoga experience (range: 6 months to 10+ years) at the time of the study (see Table 1). Several participants also reported meditation experience (*n* = 6). Participants in the RA group participated in team-based recreational sports for at least one to three 30- to 60-min sessions per week, reported less than 6 months of lifetime yoga or meditation experience, and were not practicing yoga or meditation on a regular basis at the time of the study (see *Yoga Practice* in Table 1).

**Table 1:** Participant characteristics.

Group	Gender		Age (years) Mean ± SD	Yoga Practice *(mins/week*) Mean ± SD
YP	Male	3	35.9 ± 11.5	219.0 ± 185.9
	Female	16		
RA	Male	6	32.9 ± 9.14	0
	Female	6		

*The Yoga practitioner (YP) group practiced yoga for an average of 219.0 min per week*.

### Questionnaires

Participants completed a battery of questionnaires prior to MRI testing. These questionnaires were used to characterize participants, as well as for subsequent correlational analyses. First, participants provided information as to the intensity, minutes per week, sessions per week and number of years engaged in any specific physical activities at the time of the study. These details allowed for the calculation of the METs for total physical activity completed in a week, based on a previously published compendium of MET values for various physical activities (Ainsworth et al., 2011). Next, the Mindful Attention Awareness Scale (MAAS) was self-administered to characterize groups on levels of mindfulness (Brown and Ryan, 2003). The MAAS is a validated measurement tool that indexes the frequency in which a person demonstrates mindfulness during day-to-day life (MacKillop and Anderson, 2007; Li and Goldsmith, 2012). To classify participants’ day-to-day experiences of stress over the past month, Cohen’s Perceived Stress Scale (PSS) was used (Cohen et al., 1983). This validated tool measures how stressful individuals appraise daily situations to be (Cohen et al., 1983). Finally, the Relaxation Inventory, a validated, self-administered tool comprised of three scales, measuring participants’ levels of day-to-day relaxation (psychological) and tension (physiological), was employed (Crist et al., 1989). A total, summated, score was calculated for relaxation, with higher composite scores indicating greater day-to-day relaxation. See Table 2 for group-level questionnaire results. After completing the above questionnaires participants proceeded to the imaging component of the study.

**Table 2:** Questionnaire data.

Questionnaire	YP (Mean ± SD)	RA (Mean ± SD)	Metric	*F*-test	*p*- value
Physical activity profile	4150.7 ± 2238.43	7414.9 ± 5419.82	METS/week	5.498	0.026*
Yoga activity profile	720.5 ± 621.58	N/A	METS/week		
MAAS	4.51 ± 0.66	3.8 ± 0.60	Mean Score	8.027	0.008**
PSS	10.7 ± 5.99	13.1 ± 6.08	Total Score	1.116	0.30
Relaxation Inventory	167.6 ± 20.09	152.5 ± 19.00	Total Score	4.316	0.047*

Metabolic Equivalent of Task (MET); Mindfulness Attention Awareness Scale (MAAS); Perceived Stress Scale (PSS); Yoga Practitioner (YP); Recreational Athlete (RA). *Significant at p_uncorrected_ < 0.05; **Significant at p_corrected_ < 0.0167.

### Task Design

The present study utilized a task-based block design during fMRI acquisition. We utilized a non-directive emotion-eliciting task, involving presentation of visual stimuli. During fMRI scanning, film clips were shown to evoke an emotional response in participants. To induce dichotomous emotional vs. non-emotional (control) conditions, two categories of film clips were selected: emotion-evoking (EE) and emotion-neutral (EN). EE clips contained content intended to evoke the emotions of happiness, sadness, or anger. Six film clips were chosen on the basis of validation from previous studies (Schaefer et al., 2010; Dunn and Hoegg, 2014); and two film clips corresponded to each emotion. These included scenes from the films “I am Sam” (2001) and “The Champ” (1979; sadness); “Schindler’s List” (1993; two clips for anger); and clips of people laughing (two clips for happiness). To increase the likelihood of evoking an emotional response, each participant underwent a pre-MRI screening and sensitizing period. Briefly, participants viewed trailers of each film, as well as the segment of the film that preceded the EE clip that would be viewed during the task-based fMRI. During the sensitizing period participants were instructed to focus on the emotions they felt while viewing each trailer and clip. During the task-based fMRI, the EE clips were played with no sound. EN clips were silent nature scenes (Lane et al., 2009). Like the EE clips, there were six EN clips in total. Each of the EE and EN clips were 1 min in duration; and the order of EE and EN clips was counterbalanced, while the order of each respective within-condition (i.e., EE, EN) film clip was randomized for each participant. There was a total of 12 clips separated into two functional runs. Film clips were digitally back-projected onto a screen at the rear of the MR scanner. Participants viewed the projector by looking at a mirror mounted on the head coil, within the scanner. To ensure the emotional stimuli presented were purely visual, there was no sound for the EE or EN scenes during the MR scan. Immediately after the presentation of each film clip, participants self-rated their experience of the emotions happiness, sadness and anger, each on a separate 0–8 visual analog scale (VAS; Lane et al., 2009). Briefly, participants were instructed that a rating of 0 corresponded to no emotional response, while a rating of 8 indicated a very strong emotional response.

In the MR scanner, participants were equipped with a four-lead non-ferrous ECG (Philips Healthcare, Andover, MA, USA), which was positioned as per manufacturer instructions. ECG preparation involved removing chest hair by shaving (if necessary), cleaning the skin surface with abrasive skin-preparation gel (Nuprep Skin Prep Gel, D.O. Weaver and Co., Aurora, CO, USA) and a gauze pad and thoroughly drying the skin surface with an additional gauze pad.

### MRI Acquisition

A Philips Achieva 3.0 T whole-body MR scanner (Philips Healthcare, Andover, MA, USA), with an 8-channel sensitivity encoding head coil (SENSE factor = 2.4) and parallel imaging, was used for all MRI procedures. The following scans were collected during the scanning protocol: (1) high-resolution *T*_1_-weighted anatomical scan (TR = 7.4 ms, TE = 3.7 ms, flip angle *θ* = 6°, FOV = 256 mm, 160 slices, 1 mm thickness, scan time = 3.2 min); and (2) task-based fMRI data were collected as echo-planar images, using a single-shot, blipped gradient-echo echo-planar pulse sequence (TE = 30 ms, TR = 2.0 s, flip angle *θ* = 90°, FOV = 256 mm). Two 10-min runs of fMRI data were collected. Each run contained three, 1-min periods of EN clips (30 volumes per video clip, 90 volumes total) and three, 1-min periods of EE clips (30 volumes per video clip, 90 volumes total).

### HR Data Acquisition

All HR data were collected during functional imaging runs using an ECG apparatus integrated with the MR scanner (Philips Healthcare, Andover, MA, USA). The HR intervals were 1 min each in duration. ECG was sampled at a frequency of 500 Hz.

### Data Analyses

#### fMRI Analyses

Functional MRI data were processed and analyzed using statistical parametric mapping (SPM8) software (Wellcome Department of Cognitive Neurology, University College London, UK). Images were realigned and normalized using the SPM templates. The normalized images were 3 mm × 3 mm × 3 mm and smoothed with a FWHM 8 m Gaussian kernel. Initially, a subject-wise, first-level analysis was computed using a general linear model with hemodynamic response function modeled to the fMRI task design for EE and EN conditions. Statistical parametric maps of the *t*-statistic were computed from one-sample *t*-tests for all participants for the contrast of interest, EE minus EN (EE − EN). Next, the contrast images (EE − EN) from the first-level analysis were used for the group, second-level, *t-tests*. The EE − EN contrast images were used to perform two independent *t*-tests between YP and RA groups (YP − RA and RA − YP). In a secondary analysis, mean HF-HRV was implemented as a covariate of interest in the second-level group analysis. To identify clusters of significant activation, a minimal cluster size of 20 was used and voxel-wise threshold of *p* < 0.005, uncorrected for multiple comparisons (Lane et al., 2009).

#### HR Analysis

The mean values for HR (beats per minute, bpm) and R-R interval (time between heartbeats, measured in ms) during each 1-min epoch were calculated and averaged across conditions (EE, EN), separately. This analysis, which derived HRV, was performed using the Kubios software platform (Tarvainen et al., 2014; version 2). In short, HR data underwent auto-regressive spectral analyses to generate the power spectrum of the cardiac cycle for each 1-min data collection epoch (Task Force, 1996). Two frequency bands of the HR power spectrum were analyzed: low-frequency (0.04–0.15 Hz), and high-frequency (0.15–0.4 Hz; Montano et al., 1994; Tarvainen et al., 2014). Low-frequency HRV (LF-HRV) provides a measure of SNS activity; sympathetic influence on the heart is slow and these effects are on the timescale of seconds (Montano et al., 1994). HF-HRV provides a measure of PNS activity, as these effects produce rapid beat-to-beat changes of the heart, indexing the PNS-modulated emotion regulation of stress (Lane et al., 2009). A LF/HF-HRV ratio was also calculated to estimate the SNS-PNS interaction (Perini and Veicsteinas, 2003). Artifacts were detected and removed using both visual inspection methods and Kubios standard artifact correction.

#### Statistical Analyses

For all statistical tests below, the significance level was set at *p* ≤ 0.05. In the event of a statistically significant outcome, the Bonferroni correction was used to correct for multiple comparisons, where there were greater than two levels of a dependent factor/variable of interest. Due to the unequal sample sizes, independent *t*-tests were performed, and Levene’s test was used to evaluate the equality of variances. Data were reported using mean (M), and standard deviation (SD) and mean difference (MD) and standard error (SE).

##### Behavioral Measures

An independent *t*-test was performed with METs from the physical activity questionnaire as the dependent measure to compare groups (YP, RA).

Independent *t*-tests were performed across groups (YP, RA) on outcome measures from the questionnaires (relaxation score, mindfulness score, and perceived stress score); these were corrected for multiple comparisons.

Separate independent *t*-tests were performed on VAS from ratings of happiness, sadness and anger for the EE conditions to compare groups (YP, RA) and were corrected for multiple comparisons.

##### HR Measures

Separate independent *t*-tests (Factor = Group: YP, RA) were performed on HR measures individually (mean HR, HF-HRV and LF/HF-HRV ratio) for the EE condition.

In addition, exploratory correlational analyses were performed. Pearson’s correlational analyses (*r*) were performed between brain areas of significant activation, HF-HRV during the EE condition, and Yoga METs in the YP group. Pearson’s correlational analyses (*r*) were also performed between brain areas of significant activation, HF-HRV during the EE condition, and MAAS score in the YP group. Seven correlations were performed in total. These correlational analyses were exploratory in nature, and intended to generate future hypotheses. As such, no corrections for multiple comparisons were performed.

## Results

### Questionnaire Data

Results from baseline questionnaire data are reported in Table 2. Based on METs of activity achieved per week, participants in the RA group (*M* = 7414.18, *SD* = 5419.81) tended to be engaged in more physical activity than those of the YP group (*M* = 4150.73, *SD* = 2238.42); however, this difference did not reach significance [*t*_(1,13.4)_ = 1.982, *p* = 0.068, 95% CI (−282.45, 6810.78)].

Additionally, after correcting for multiple comparisons (Bonferroni corrected alpha of levels of 0.0167), there was a significant difference for day-to-day depositional mindfulness (MAAS scoring) across groups [*t*_(1,29)_ = 2.83, *p* = 0.008, 95% CI (−1.15, −0.18)], whereby the YP group scored higher than the RA group (4.51 ± 0.662 > 3.84 ± 0.603). With regards to the PSS, there was no significant difference between RA group (*M* = 13.08, *SD* = 6.08) and YP group [*M* = 10.73, *SD* = 5.98; *t*_(1,29)_ = 1.05, *p* = 0.30, 95% CI (−2.20, 6.89)]. Lastly, there was a non-significant trend (after correcting for multiple comparisons) for group differences in relaxation [Relaxation Inventory scoring: MD = −15.08, *SE* = 7.26; *t*_(1,29)_ = 2.07, *p* = 0.047, 95% CI (−29.92, −0.23)]. Specifically, the YP group tended to score higher than the RA group on the Relaxation Inventory (167.58 ± 20.09 > 152.5 ± 19.00).

### Emotion Ratings

After correcting for multiple comparisons, there were no significant differences in VAS scores between groups for ratings of happiness, sadness and anger for the respective corresponding film clips (*t*_(1,29)_ < 0.93, *p* > 0.36; Table 3).

**Table 3:** Visual analog scale (VAS) data.

VAS	YP (Mean ± SD)	RA (Mean ± SD)	*p* value
Happy	5.31 ± 0.92	5.46 ± 1.21	0.71
Sad	4.13 ± 1.57	4.67 ± 1.51	0.36
Anger	4.79 ± 2.02	5.33 ± 1.68	0.44

*Group ratings for the emotions of Happy, Sad and Anger following corresponding emotion-evoking (EE) conditions; Yoga Practitioner (YP); Recreational Athlete (RA)*.

### HR Measures

**Figure 1:**
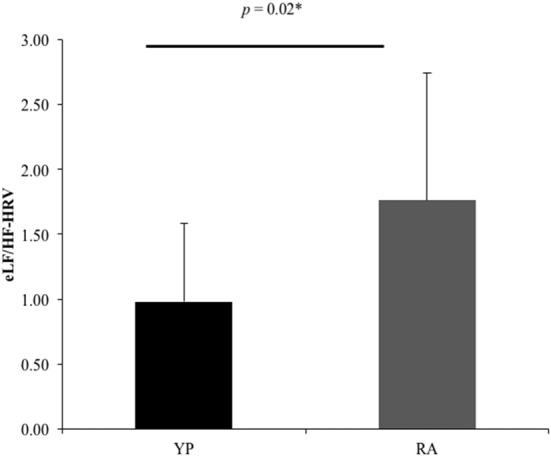
There was a significant difference between groups for the ratio between low-frequency and high frequency-heart rate variability (eLF/HF-HRV; *U* = 7.57, *p* = 0.02). *Significant at p_uncorrected_ < 0.05.

Multiple comparisons were accounted for using a Bonferroni-corrected alpha level of 0.0167. There was a nonsignificant trend for a Group effect in LF/HF-HRV ratio for the EE condition, with the YP group (*M* = 0.98, *SD* = 0.60) showing lower scores as compared to the RA group [*M* = 1.76, *SD* = 0.98; *t*_(1,16.36)_ = 2.47, *p* = 0.025, 95% CI (0.11, 1.45); Table 4, Figure 1]. Otherwise, there were no significant differences between groups for mean HR or HF power in the EE conditions (*t*_(1,29)_ < 1.48, *p* > 0.15).

**Table 4:** Heart rate data.

HR measure	YP (Mean ± SD)	RA (Mean ± SD)	Test	*p* value
eHR	66.0 ± 8.57	61.6 ± 7.20	ANOVA	0.15
eLF/HF-HRV	0.98 ± 0.61	1.76 ± 0.98	Mann-Whitney	0.02*
eHF-HRV	916.0 ± 580.13	746.2 ± 502.13	Mann-Whitney	0.35

Heart rate in the emotion conditions (eHR); low-frequency to high frequency ratio heart rate variability in the emotion and neutral conditions (eLF/HF-HRV); high frequency heart rate variability in the emotion condition (eHF-HRV); Yoga Practitioner (YP); Recreational Athlete (RA). *Significant at p_uncorrected_ < 0.05.

### Brain Activation During EE Condition

**Figure 2:**
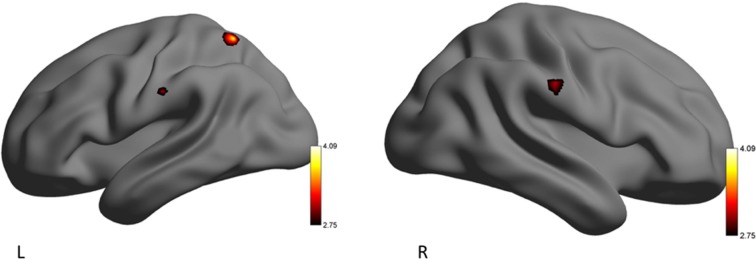
Left (L) and right (R) views of areas demonstrating significant activation during the emotion-evoking (EE) condition for the yoga practitioners (YP) > recreational athletes (RA) contrast. The color scale reflects *t*-values.

To counter low statistical power, emotion conditions (eHR) were collapsed to produce global “EE” vs. “EN” conditions. When emotional conditions (happiness, sadness, anger) were collapsed, the YP group activated clusters of voxels in the left superior parietal lobule, postcentral gyrus and right anterior supramarginal gyrus. In the secondary analysis, there were no additional significant clusters of activation when HF-HRV was entered as a covariate of interest (Table 5, Figure 2).

**Table 5:** Clusters demonstrating significant for the second-level EE contrast: YP minus RA.

Region	T	X	Y	Z	mm^2^
Left superior parietal lobule	4.09	−30	−55	58	34
Left postcentral gyrus (supramarginal gyrus)	3.69	−51	−22	28	17
Right supramarginal gyrus (anterior division)	3.32	60	−19	34	23

**Figure 3:**
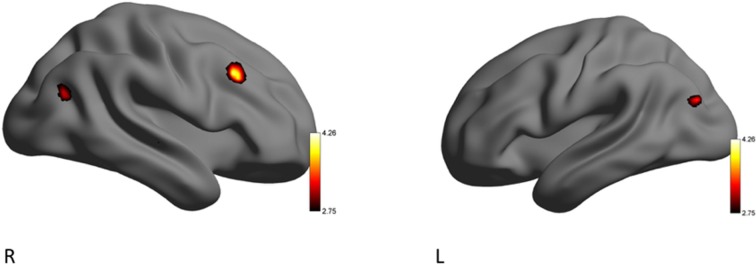
Left (L) and right (R) views of areas demonstrating significant activation during the EE condition for the RA > YP contrast. The color scale reflects *t*-values.

The RA group activated clusters of voxels in the right middle frontal gyrus, and lateral occipital cortex when eHR (happiness, sadness, anger) were collapsed. In the secondary analysis, there were no additional significant clusters of activation when HF-HRV was entered as a covariate of interest (Table 6, Figure 3).

**Table 6:** Clusters demonstrating significant for the second-level EE contrast: RA minus YP.

Region	T	X	Y	Z	mm^2^
Right inferior frontal gyrus	4.35	42	20	34	68
Right lateral occipital cortex	3.91	36	−64	22	71

### Relationships Between Questionnaire Outcome Measures and HR Measures

**Figure 4:**
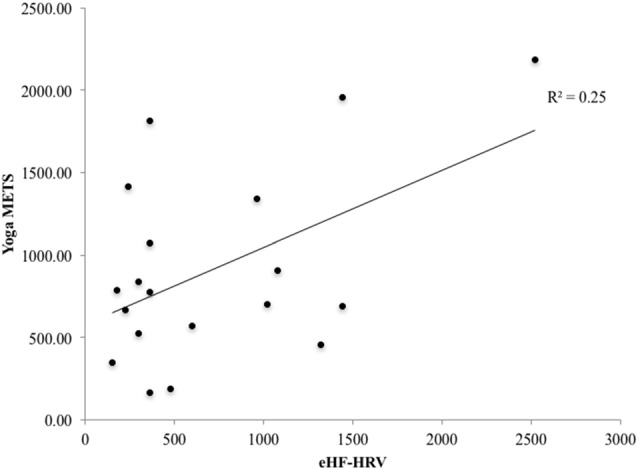
There was a trend towards a significant relationship for the YP group between the number of the yoga metabolic equivalent of task (MET) calculated from the physical activity profile questionnaire and high frequency heart rate variability (HF-HRV) during the emotion conditions (*r* = 0.43, *p* = 0.067).

There was a nonsignificant trend for a relationship between total METs per week engaged in yoga, and HF-HRV during the EE condition (*r* = 0.43, *p* = 0.067; Figure 4). This trend suggests that greater time and intensity of yoga practice may be associated with higher HF-HRV during EE film viewing.

In the YP group, there were no significant relationships between brain activity (left superior parietal lobule, postcentral gyrus and right anterior supramarginal gyrus) associated with the EE condition and the total METs per week engaged in yoga (*r* < 0.22, *p* > 0.36).

There were no significant relationships in the YP group between MAAS rating score and brain activity (left superior parietal lobule, postcentral gyrus and right anterior supramarginal gyrus) associated with the EE condition (*r* < 0.30, *p* > 0.21).

## Discussion

The present study investigated the effects of yoga experience on the neurovisceral components (HRV and fMRI) of emotion regulation using a cross-sectional sample of yoga practitioners (YP) and physically active individuals not practicing yoga (RA). Our primary aim was to determine whether YP participants exhibited different patterns of brain activation compared to RA participants, while viewing emotionally arousing visual stimuli. We hypothesized that during observation of emotional stimuli YP participants would demonstrate significantly different patterns of brain activity in regions that have been implicated in emotion regulation, as compared to RA participants. Our secondary aim was to examine potential differences across groups in HRV throughout the presentation of these stimuli. Here, we hypothesized that YP participants would exhibit significantly greater HF-HRV, compared to RA participants, during the emotional stimuli.

Differences in patterns of brain activity were observed during times of emotion regulation (EE video clips) between individuals experienced in the practice of yoga (YP group) and recreational athletes with little-to-no previous, and no ongoing, yoga experience (RA group). Based on the findings from the present study, we show that yoga experience may enable the use of adapted neurophysiological processing during situations that demand emotion regulation. During the EE condition RA participants demonstrated brain activity in the frontal cortex, similar to beginner meditators who experienced emotional stimuli (Taylor et al., 2011). In contrast, while there was no significant difference between subjective ratings of emotional experience, YP participants activated the superior parietal lobule, postcentral gyrus and supramarginal gyrus, during the EE conditions. These observations reflect patterns of brain activation exhibited by expert meditators during emotional situations (Lutz et al., 2008; Taylor et al., 2011). While there were no unique areas of brain activation that significantly correlated with HF-HRV for either group during the EE condition, the YP group tended to maintain greater parasympathetic control as demonstrated by a trend towards a lower ratio of LF/HF-HRV during the EE conditions. Reduced activation of the frontal cortex and a lower LF/HF-HRV ratio may demonstrate that individuals who practice yoga differently regulate emotional stimuli. Consequently, the YP participants showed a differential neural pattern of emotional processing via activation of their right supramarginal gyrus (Silani et al., 2013), compared to RA participants whose greater activation of their right middle frontal gyrus was reflective of individuals with lesser emotional stability (Taylor et al., 2011). These findings, from a relatively small sample of individuals (YP: *n* = 19; RA: *n* = 12), demonstrate an important need for future studies to investigate emotion regulation amongst populations of individuals that engage in physical and cognitive practices, such as yoga.

### HRV During Emotion Regulation

The YP group had marginally but not significantly increased HF-HRV and decreased LF-HRV during both EE and EN conditions compared to the RA group, as reflected by the trend towards a lower LF/HF-HRV ratio relative to the RA group (Table 1). A lower LF/HF-HRV ratio has been shown to reflect decreased SNS activity, increased PNS activity, or both (Perini and Veicsteinas, 2003). Changes in resting-state autonomic balance have been observed following 4 weeks of yoga practice, with greater shifts towards PNS dominance, potentially signifying increased regulation of stress (Patil et al., 2013). Similar results have also been shown in persons who underwent a 5-month yoga intervention, compared to a control group (Nagendra et al., 2015), as well as within a 5-day mind-body training program, compared to a relaxation group (Tang et al., 2009). The present study augments these findings, suggesting that long-term, consistent yoga practice may benefit the regulation of stress not only during rest, but also in situations where emotion regulation is necessitated.

In the present study, recreational athletes were recruited as controls for our YP group due to the known influence of physical activity and increased cardiovascular fitness on autonomic function (Hautala et al., 2009). While cardiovascular fitness influences HRV, in a group of 103 healthy men and women, Dishman et al. (2000) found perceived stress to be the strongest predictor of LF-HRV, independent of age, gender, trait anxiety and cardiorespiratory fitness level (Dishman et al., 2000). The present study did not show a significant group difference for level of perceived stress for the previous month. Therefore, future work is needed to determine the effects of physical activity participation on HRV during emotional-evoking situations.

### Neural Correlates of Emotion-Eliciting Stimuli

The fMRI data from the present work further support the observations from the HRV data, suggesting that YP participants activated a unique set of brain regions that permit dynamic autonomic control over the heart. During the EE condition, the RA group activated a brain region commonly observed during situations that require emotion regulation (Thayer et al., 2012). The middle frontal cortex is associated with the conscious control of emotions, sending reciprocal signals to down-regulate emotional responses and integrate autonomic responses (Thayer et al., 2012; Frank et al., 2014). Specifically, increased activation in the middle frontal lobe indicates that the RA group may have participated in cognitive reappraisal of their emotions (Silani et al., 2013). This result is analogous to evidence from electroencephalography (EEG) work showing that experienced meditators exhibit significantly lower frontal activation in the late positive potential (LPP) event-related potential (ERP), in response to negative emotional images, vs. a control group with no meditation experience (Sobolewski et al., 2011). In accordance with previous studies, the RA group, may rely on frontal cortex activation to facilitate emotion down-regulation processes similar to beginner meditators (Brefczynski-Lewis et al., 2007; Taylor et al., 2011). Indeed, two meta-analyses exclusively looking at neural activation during cognitive reappraisal of emotion found right middle frontal gyrus as a key part of the neural system (Diekhof et al., 2011) and emotion down-regulation (Frank et al., 2014). Interestingly, in a recent study, Bernstein and McNally (2017) found that an acute bout of aerobic exercise attenuated negative emotions (evoked by film clips) in a physically active group of young healthy adults who experienced difficulties with emotion regulation (i.e., feeling stuck in an induced mood state). The authors suggested that, in this group of individuals, exercise may have helped down-regulate negative emotions through regulatory strategies and goal-directed cognition and behavior, compared to a control group who did not participate in exercise (Bernstein and McNally, 2017). While neurophysiological mechanisms were not investigated, and the type of physical activity was not reported for the participants, the proposed emotion regulation strategies support the present findings, demonstrating that young healthy individuals who participate in regulate physical activity may engage in cognitive-based emotion regulation.

### Yoga Practice and Associated Neurophysiology

Unlike the RA group, the YP group demonstrated activation of a unique set of brain regions that may reflect an adapted means of emotion regulation that emphasizes less conscious regulatory processing. Specifically, the largest cluster was observed in the left superior parietal lobule, which is a part of attention and executive control networks, coordinating attention under competing conditions and voluntary orienting of attention (Corbetta and Shulman, 2002). Yoga demands a high attentional state as postures are held for sustained periods of time. During yoga practice, individuals are instructed to consciously control their breathing rate and depth, as well as body alignment and position and emotional state (Villemure et al., 2015). Attention regulation has been positively linked to emotion regulation (Wadlinger and Isaacowitz, 2011), and therefore the increased involvement of the superior parietal cortex may explain the YP group’s ability to selectively attend to their internal and external environment resulting in enhanced parasympathetic control and emotion regulation processes.

The YP group, unlike the RA group, significantly activated the supramarginal gyrus, which is also a part of the sensorimotor network. Recently, the right supramarginal gyrus was associated with feelings of empathy and overcoming emotional egocentric biases, during emotionally arousing conditions (Bernhardt and Singer, 2012; Silani et al., 2013; Steinbeis et al., 2015). Other work has indicated that parietal brain regions may be key neurophysiological components in the unattached moment-to-moment awareness of emotions (Marchand, 2014). Combined with recent evidence on the role of the supramarginal gyrus, the present findings indicate that YP participants appear to have dispassionately attended to the emotions within the EE film clips, as opposed to presenting an egocentric perspective. Thus, the practice of yoga, which purports to teach individuals to cultivate compassion and empathy (Hofmann et al., 2011), may have modulated participants’ emotion regulation process, as demonstrated by a trend towards a lower ratio of SNS-to-PNS activity in the YP group.

### Limitations and Future Directions

Due to the small sample size in the present study (*n* = 31), we were unable to investigate sex or gender differences in emotion regulation within and between our groups (YP, RA). Due to the known differences between females and males during emotion regulation (McRae et al., 2008), future studies with larger sample sizes should examine sex or gender-dependent brain activation between individuals who participate in a regular yoga practice and those who do not. Our small sample size and unbalanced groups (YP, *n* = 19; RA, *n* = 12) resulted in reduced power, and an increased likelihood of false negative findings. To further understand the effect of yoga practice on emotion regulation, future studies should be more adequately powered to better investigate neurophysiological responses to different types of emotion-eliciting stimuli. Indeed, due to our sample size we collapsed our analyses across emotion-eliciting stimuli (happy, sad, anger). Differences in areas of brain activation have been observed for happy compared to sad stimuli (Harada et al., 2016), and therefore, further distinctions between HRV and fMRI measures may be present between YP and RA groups for different evoked emotions. Similarly, it would be interesting to examine how separate positive and negative emotions are centrally processed by those with varying degrees of yoga experience.

Due to previously observed discrepancies between self-reported and neurophysiological responses to emotional stimuli (Connelly and Denney, 2007; Nandrino et al., 2012; Shepherd and Wild, 2014), we did not measure psychological strategies, such as the level of state mindfulness, or actual emotion regulation behavior, during the viewing of the emotional stimuli. Therefore, interpretations of our findings are strongly based on previous observations from the emotion regulation literature. In the future, to increase our understanding of connection between behavioral approaches and neurophysiological responses to emotion regulation, subjective ratings of emotional response, measurements of emotion regulation strategies (acceptance, mindfulness, suppression and endurance; Wilson et al., 2014), and neurophysiological methods should be investigated in conjunction.

Based on patterns of activation previously reported in expert meditators, the reduced frontal activation in participants from the YP group as opposed to the significant frontal activation in the RA group may reflect greater emotional stability (Taylor et al., 2011). We believe these results to be in line with existing regulatory mechanisms of yoga, as individuals who practice yoga appear to have a greater ability to attend to what is taking place in the present, through a process of non-appraisal while achieving a sustained awareness of ongoing events and experiences without evaluation or judgment (Schmalzl et al., 2015).

## Conclusion

Functional MRI and HRV analyses are an effective technique to assess the influence of yoga practice on emotion regulation strategies during a non-directive emotion-eliciting task. The present findings support the notion that yoga experience uniquely modulates the activity of several brain regions that are central to emotion regulation, and results in a slightly diminished sympathetic (or heightened parasympathetic response) to potentially stressful emotional stimuli compared to recreational athletes. Accordingly, yoga experience may allow individuals to: (1) regulate the emotion generation process through greater flexibility, acceptance and non-attachment to the self when observing or experiencing emotions; and (2) be more empathic when presented with an emotional situation, by attending to the emotions of other individuals. These observations are reflected in lower frontal cortical activity, and activation of the supramarginal gyrus in the YP group, as opposed to those individuals in the RA group. As stated by Bernstein and McNally (2017), the investigation into the therapeutic mechanisms of physical activity on emotion regulation is in its infancy, and the findings from the present study support the need for further investigation into the beneficial effects of different forms of physical activity on emotional health. In conclusion, the practice of yoga may help individuals learn to accept and experience emotions, and to acquire the ability to separate their own emotions from those of others. Such a shift in emotion regulation may indicate that the practice of yoga could present a means for improving psychological and physical health.

## Author Contributions

KW, NS, PS, SS, TW and LB conceived and planned the present study and contributed to the interpretation of the results. KW and NS were responsible for participant recruitment and data collection. KW, NS and LB were responsible for data analysis. KW, NS and PS wrote the manuscript. All authors provided critical feedback on the manuscript.

## Conflict of Interest Statement

SS and TW were employed by company lululemon athletica. The remaining authors declare that the research was conducted in the absence of any commercial or financial relationships that could be construed as a potential conflict of interest.
